# Herpes Simplex Virus Meningoencephalitis Masquerading as Acute Stroke With Broca's Aphasia: A Case Report

**DOI:** 10.7759/cureus.40618

**Published:** 2023-06-19

**Authors:** Gagandeep Singh Arora, Don Phung, Parneet Kaur

**Affiliations:** 1 Internal Medicine, University of California, Riverside, School of Medicine, Riverside, USA; 2 Internal Medicine, Suburban Community Hospital, Norristown, USA

**Keywords:** broca's aphasia, herpes simplex virus, acyclovir therapy, lumbar puncture, mri findings, diagnostic challenge, stroke mimic, expressive aphasia, meningoencephalitis, herpes simplex virus (hsv)

## Abstract

This case report presents a rare and unique instance of a 70-year-old morbidly obese female with type 2 diabetes mellitus and bilateral lymphedema, who presented with fever and expressive aphasia, initially suspected to be a stroke. A negative CT scan prompted the performance of an MRI, which revealed suggestive imaging findings of herpes encephalitis. Following the MRI, the patient experienced seizures and required intubation in the intensive care unit. Subsequently, a lumbar puncture was performed, confirming the diagnosis of herpes simplex virus (HSV) meningoencephalitis. Prompt initiation of acyclovir therapy led to an improvement in aphasia, ultimately allowing for extubation and transfer to the general ward. The rarity of this case lies in the unusual manifestation of Broca's aphasia caused by HSV, which is not typically associated with this neurological deficit. This report highlights the importance of considering herpes encephalitis as a potential etiology in patients presenting with atypical neurological symptoms, even in the absence of typical radiological findings. Early diagnosis and appropriate management with acyclovir are crucial in improving outcomes in such cases.

## Introduction

Herpes simplex virus type 1 (HSV-1) is the most common cause of life-threatening sporadic encephalitis across the globe [1] and does not exhibit any seasonal variation [1]. Herpes simplex virus (HSV) meningoencephalitis is a serious infection affecting the brain and its surrounding membranes, which can present with various symptoms such as high fever, severe headaches, altered mental status, seizures, and focal neurological deficits. Temporal lobe involvement is observed in 60% of patients, with pure temporal lobe involvement in 20% and pure extratemporal involvement in 15% [1]. These deficits can include weakness or paralysis in particular body parts, sensory abnormalities, coordination problems, and visual disturbances. Speech difficulties such as slurred speech or difficulty finding words are also common and may manifest in various ways such as dysarthria, expressive or receptive language difficulties, or aphasia [1].

Aphasia refers to a communication disorder that is characterized by difficulty for a person to use words to exchange information. It is divided into expressive aphasia and receptive aphasia, with expressive aphasia also being called Broca's aphasia. The main cause of Broca's aphasia is often a stroke that affects the dominant inferior frontal lobe or Broca area of the brain (Brodmann area 44 and 45) [2]. Other etiologies such as traumatic brain injury, tumors, infections, and degenerative dementing illnesses can also contribute to the development of Broca aphasia. Broca area is responsible for translating thoughts into words, so when such aphasia occurs, individuals may struggle to express their thoughts and experience difficulties with speech fluency [2].

In this case report, we present a patient with fever and exclusively expressive aphasia. In addition to medical treatment, speech therapy and rehabilitation are often recommended to help individuals improve their communication abilities. Prompt recognition and management of focal neurological deficits are crucial for the effective treatment of HSV meningoencephalitis.

## Case presentation

The patient was a right-handed 70-year-old female with a past medical history of chronic lymphedema, type 2 diabetes, hypertension, and no previous history of seizures, surgeries, or significant family history. She presented to the emergency department with complaints of a two-day history of confusion and fever.

On her initial presentation, she was febrile with a recorded temperature of 101.4°F. Her physical exam revealed that she had severe difficulty with speaking and had trouble with word finding. She was able to comprehend our questions but was unable to find words to express herself. She answered questions by nodding from side to side for no and up and down for yes. She was alert and oriented to place and person only, and had normal muscle strength with grossly intact cranial nerves. No signs of headaches, neck stiffness, nausea, or vomiting were present. The initial workup with a CT scan of her head was without any abnormalities. Urinalysis was consistent with UTI. Her blood investigations are mentioned in Table 1.

**Table 1:** Blood investigations mEq/L: milliequivalents per liter; mg/dl: milligrams/deciliter; mmol/L: millimoles/liter; U/L: units/liter; IU/L: international units/liter.

Investigation	Value	Reference range
Hemoglobin	12.4	12-16 g/dl
Total leucocyte count	12,600	4.5-11 cells/microliter
Glucose	139	120-140 mg/dl
Creatinine	0.7	0.6-1.1 mg/dl
Sodium	139	135-145 mEq/L
Potassium	4.8	3.5-5.5 mEq/L
Chloride	90	96-106 mEq/L
Ammonia	20	11-32 micromol/L
Anion gap	8	4-12 mmol/L
Aspartate aminotransferase	35	8-33 U/L
Alanine aminotransferase	14	4-36 U/L
Alkaline phosphatase	65	44-147 IU/L
Total bilirubin	0.4	0.1-1.2 mg/dl

She was initiated on Tylenol, intravenous fluids, and Ceftriaxone empirically for infectious sources. MRI was ordered to evaluate the etiology of her expressive aphasia further as she had the risk factors for stroke (obesity, hyperlipidemia, and diabetes). Right after she was shifted to the ward from MRI, she had a generalized tonic-clonic seizure. She was then transferred to the intensive care unit after intubation for the protection of her airway.

**Figure 1:**
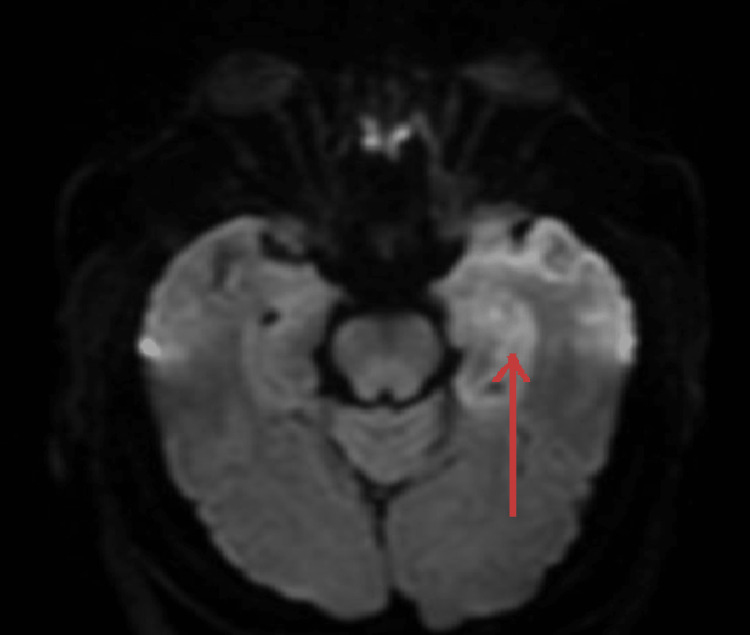
MRI scan, diffusion-weighted imaging phase MRI of the patient showing restricted diffusion involving the left temporal lobe and left insular cortex.

**Figure 2:**
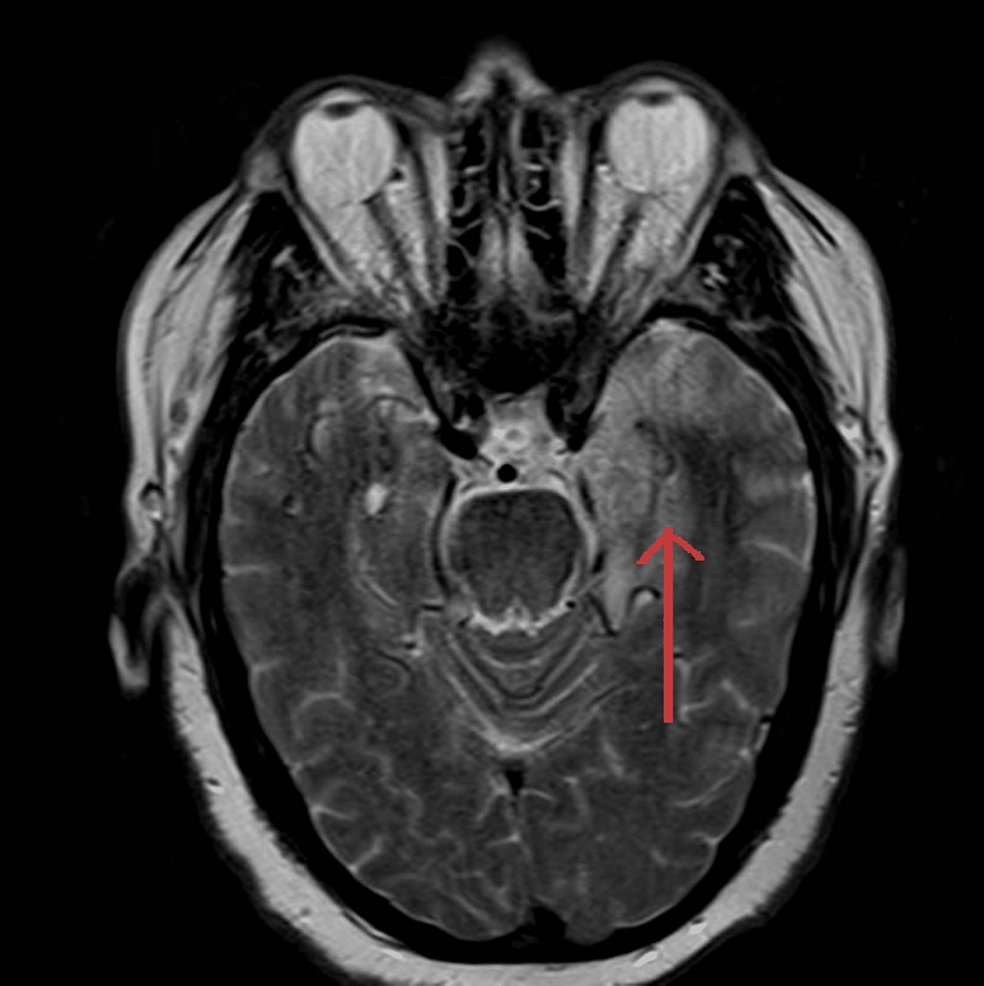
MRI scan, T2-weighted imaging phase Abnormal high signal on T2 involving the left insular cortex and left temporal lobe. No intra- or extra-axial hemorrhage was seen.

**Figure 3:**
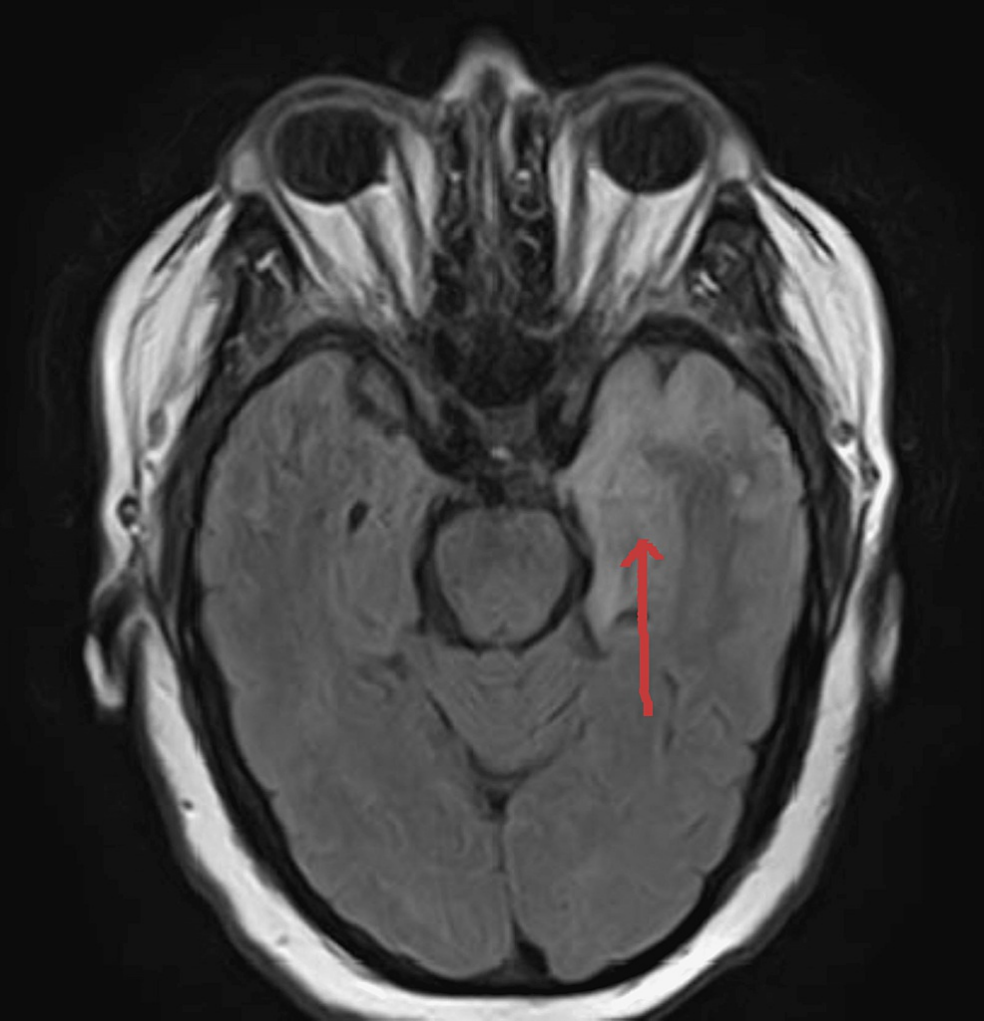
MRI scan, FLAIR phase An abnormal high signal was seen on FLAIR involving the left insular cortex and left temporal lobe. FLAIR: fluid-attenuated inversion recovery.

MRI report came out subsequently, which showed restricted diffusion involving the left temporal lobe and left insular cortex. Differential diagnoses included left middle cerebral artery distribution infarct versus encephalitis such as herpes encephalitis (Figure 1). An abnormal high signal was seen on T2 (Figure 2) and fluid-attenuated inversion recovery (FLAIR; Figure 3) involving the left insular cortex and left temporal lobe.

CT angiography of the head and neck with contrast was done, which showed no evidence of hemodynamically significant stenosis, acute thrombosis, dissection, or aneurysm.

Given the MRI findings and suspicion of meningoencephalitis, the intensivist decided to perform a lumbar puncture in the ICU to rule out meningitis and initiated her on ampicillin, vancomycin, and acyclovir.

The CSF was sent for cytology, serology, and culture. CSF findings (Table 2) revealed a WBC of 36 with 80% lymphocytes, glucose at 120, and protein at 90. CSF fluid revealed the presence of HSV-1 on polymerase chain reaction.

**Table 2:** CSF fluid analysis HSV: herpes simplex virus; IgG: immunoglobulin type G; mg/dl: milligrams/deciliter.

CSF studies	Value	Reference range
WBC	36	0-5 cells/microliter
Lymphocytes	80%	
Segs	11%	
Monocytes	9%	
Glucose	120	50-80 mg/dl
Protein	90	15-45 mg/dl
RBC	17	0 cells/microliter
Color	Colorless	Colorless
HSV 1/2 IgG antibody titer	2.32 IV	0.89 IV or less

Serology came back negative for antibodies against Epstein-Barr virus, West Nile virus, measles, mumps, and varicella-zoster virus. The patient was found to have raised IgG antibodies against HSV 1/2. Rest all antibodies were within normal limits. Cultures were negative as well.

The patient was hence confirmed to have herpes simplex meningoencephalitis. Acyclovir was continued along with ceftriaxone.

In two days, she was successfully extubated and was downgraded. Her symptoms improved, and she was able to answer simple questions. At the time of her discharge, she still had difficulty answering open-ended questions with residual word-finding difficulties. Upon discharge, she was continued on acyclovir with recommendations for a total 21-day course and was asked to undergo speech therapy.

## Discussion

**Figure 4:**
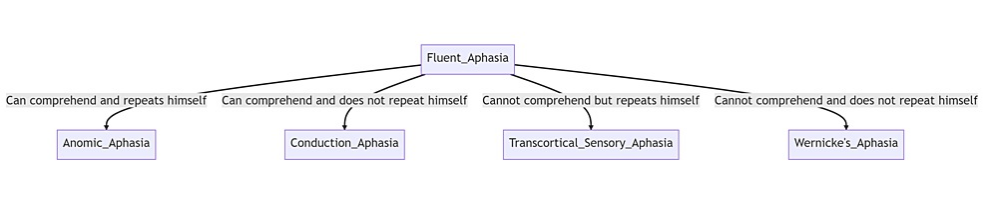
Types of fluent aphasias Flowchart made at mermaid.live

**Figure 5:**
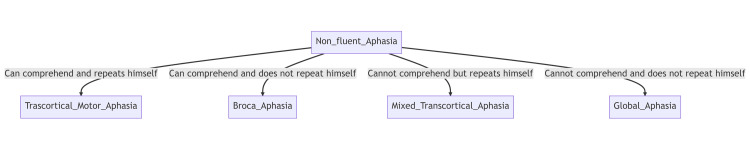
Types of non-fluent aphasias Flowchart made at mermaid.live

Aphasias can be divided into fluent and non-fluent aphasias depending on if the patient can speak in normal-sounding sentences or not and which in turn depends on the region of the brain they affect. In fluent aphasia, the patient is able to speak fluently but their speech is purposeless, and often communication includes "paraphasia," which is swapping one word (planned) in a sentence with another that is inaccurate. In non-fluent aphasia, the patient struggles to form grammatically correct speech and writing. Their speech is non-fluent, lacking prepositions, articles, and other grammatical elements [3]. Refer to Figures 4, 5 for their subtypes.

HSV can mimic stroke as the patient's manifestation can be identical to stroke presenting as a focal neurological deficit [4]. One of the rare focal neurological deficits with HSV encephalitis is aphasia. We searched PubMed with the keywords HSV and aphasia. We found 88 records out of which 10 were relevant to our case. These are mentioned below along with their presentations.

**Table 3:** Previous case reports

Case report	Type of aphasia
Win et al. (2019) [4]	Fluent aphasia
Devaraj et al. (2018) [5]	Aphasia was likely multifactorial (the patient had past history of herpes simplex virus encephalitis with Wernicke's encephalopathy and seizure disorder)
Abdelmalik et al. (2015) [6]	Global aphasia
Soares-Ishigaki et al. (2012) [7]	Mixed aphasia, impairment of short-term memory, working memory, and dyscalculia
Funayama et al. (2007) [8]	Wernicke’s aphasia, ideomotor apraxia, and limb kinetic apraxia
Khan et al. (2006) [9]	Broca aphasia
Sangermani et al. (1999) [10]	Anomic aphasia in a normally fluent context
Jibiki et al. (1992) [11]	Gogi (word-meaning) aphasia-like transcortical sensory aphasia and neologism
Van Hout et al. (1986) [12]	Wernicke’s aphasia

Wernicke's aphasia was seen most commonly with HSV encephalitis (three cases). Broca's aphasia has been previously reported only once by Khan et al. [9].

Our case of expressive aphasia (Broca's aphasia) as a result of HSV encephalitis was initially brought to attention from the results of her MRI, which was later confirmed by CSF studies from a lumbar puncture. In the case of our patient, it was likely that she presented early in the course of her infection given her grossly normal CT scan of her head.

Broca's area is present in the inferior frontal gyrus of the brain [13]. HSV encephalitis predominantly affects the temporal lobe and limbic system. Occurrence of Broca's aphasia in herpes simplex encephalitis is thus rare and its occurrence can be explained by transneuronal spread of the virus that has previously been described as well [14].

Typically, early diagnosis is difficult with CT, as it generally shows subtle low-density findings of the temporal lobe and insular cortex [15].

## Conclusions

This case emphasizes the rare manifestation of HSV meningoencephalitis presenting as expressive aphasia in a patient initially suspected of having a stroke. Prompt diagnosis, through the utilization of MRI and confirmatory lumbar puncture, led to the initiation of acyclovir therapy and subsequent improvement in aphasia. This report emphasizes the importance of considering HSV encephalitis as a differential in cases of atypical neurological deficits, highlighting the need for early recognition and appropriate management to optimize patient outcomes.
